# Association between Iron Intake and Diabetic Peripheral Neuropathy in Type 2 Diabetes: Significance of Iron Intake and the Ratio between Iron Intake and Polyunsaturated Fatty Acids Intake

**DOI:** 10.3390/nu12113365

**Published:** 2020-11-01

**Authors:** Kyuho Kim, YoonJu Song, Tae Jung Oh, Sung Hee Choi, Hak Chul Jang

**Affiliations:** 1 Department of Internal Medicine, Seoul National University Bundang Hospital, Seongnam 13620, Korea; penguinkkhneo@gmail.com (K.K.); shchoimd@gmail.com (S.H.C.); janghak@snu.ac.kr (H.C.J.); 2 Department of Food Science and Nutrition, The Catholic University of Korea, Bucheon 14662, Korea; yjsong@catholic.ac.kr; 3 Department of Internal Medicine, Seoul National University College of Medicine, Seoul 03080, Korea

**Keywords:** diabetic peripheral neuropathy, dietary intake, iron, polyunsaturated fatty acid, type 2 diabetes

## Abstract

We aimed to investigate the association of iron and polyunsaturated fatty acid (PUFA) intake with diabetic peripheral neuropathy (DPN) in individuals with type 2 diabetes. This cross-sectional study included 147 individuals with type 2 diabetes. Dietary intake was assessed using three-day food records. DPN was diagnosed on the basis of a Michigan Neuropathy Screening Instrument—Physical Examination score ≥2.5. Adjusted for total energy intake, iron intake was significantly higher in individuals with DPN than in those without DPN (10.9 ± 4.0 mg vs. 9.9 ± 3.6 mg, *p* = 0.041). In addition, the iron/PUFA ratio was significantly higher in individuals with DPN (1.4 ± 0.8 vs. 1.1 ± 0.4, *p* = 0.005). Logistic regression analyses showed that iron intake (odds ratio (OR): 1.152; 95% confidence interval (CI): 1.012, 1.311) and iron/PUFA ratio (OR: 2.283; 95% CI: 1.066, 4.887) were associated with DPN after adjustment for total energy intake, sex, age, body mass index, systolic blood pressure, diabetes duration, estimated glomerular filtration rate, glycated hemoglobin, low-density lipoprotein cholesterol, and smoking. In conclusion, high dietary iron intake and an elevated iron/PUFA ratio were associated with the presence of DPN. The present study suggests the importance of the dietary pattern of iron and PUFA intake in individuals with type 2 diabetes.

## 1. Introduction

Diabetic peripheral neuropathy (DPN) is the most common form of diabetic neuropathy [1]. It is an important cause of foot ulceration and a major contributor to falls and fractures [2]. Long-duration diabetes, old age, hyperglycemia, hypertension, dyslipidemia, obesity, alcohol, smoking, and insulin resistance are known risk factors for DPN [3,4]. From a pathophysiologic point of view, oxidative stress is a key contributor to DPN [5]. However, alpha-lipoic acid, a currently available antioxidant treatment, showed a clinically relevant effect on symptomatic DPN when administered by intravenous infusion [6]. Furthermore, there is no approved preventive or curative treatment for DPN other than risk factor management. Therefore, the identification of additional modifiable factors is crucial for developing a new strategy to treat DPN.

Diet plays a critical role in the development of type 2 diabetes and its complications [7,8]. The Mediterranean [9] and vegetarian [10] diets reduce diabetes risk. In addition, a low-carbohydrate, high-unsaturated and low-saturated fat diet improved glycemic control and cardiovascular disease risk factors [11]. Several studies showed that the blood concentration of micronutrients was decreased in individuals with DPN. For example, vitamin D deficiency is associated with DPN [12] and with painful DPN even after adjusting for confounding factors [13]. Among metformin users, vitamin B12 deficiency was commonly detected and might have a detrimental effect on DPN [14]. The blood levels of other vitamins B such as vitamin B1 and B6 [15] were also associated with a high frequency of DPN. Therefore, assessing these micronutrients might be useful for the stratification of DPN risk [16]. However, from the perspective of clinical practice, the measurement of the blood levels of micronutrients might not be feasible in terms of cost and complexity of the procedure. In this regard, nutritional assessment might be a solution to evaluate an individual’s risk of DPN.

Iron, a trace element, participates in a variety of cellular processes, including oxygen delivery, mitochondrial electron transport, DNA synthesis, and gene regulation [17]. However, excess iron can generate oxidative stress and cause tissue damage [18]. Interestingly, previous studies have shown an association between dietary iron intake and diabetes risk [19,20]. Several rodent models of DPN have shown that iron deficiency rather than iron overload was associated with the risk of DPN [21,22,23]. However, no study has evaluated the association between dietary iron intake and DPN in humans.

Polyunsaturated fatty acids (PUFA), especially, omega-3 PUFA, are antioxidants [24]. Intake of PUFA and replacement of saturated fatty acids (SFA) with PUFA reduced the risk of type 2 diabetes [25,26]. In addition, a high dietary PUFA intake was associated with a lower risk of DPN [27]. Despite these findings, few studies have evaluated the association between these nutrients and DPN. Therefore, in this study, we examined the association of iron intake and of the ratio between iron intake and PUFA intake (iron/PUFA) with DPN in individuals with type 2 diabetes.

## 2. Materials and Methods

### 2.1. Study Population

The original prospective observational study was designed to discover reliable screening tools and biomarkers for DPN in type 2 diabetes. The present study analyzed data from individuals who were enrolled during the initial 2-year period (2017–2019) of the prospective observational study at the Seoul National University Bundang Hospital (SNUBH). We recruited 200 individuals with type 2 diabetes regardless of the presence of DPN. The inclusion criteria were: age ≥19 years, diagnosis of type 2 diabetes, and no change in glucose-lowering drugs in the last 3 months. The exclusion criteria were: other causes of neuropathy such as heavy alcohol consumption (alcohol consumption >30 g/day for men and >20 g/day for women), chronic kidney disease (estimated glomerular filtration rate (eGFR) <30 mL min^−1^ (1.73 m)^−2^), pregnancy, and severe foot ulcers requiring hospital admission. The study was approved by the Institutional Review Board of the SNUBH (no. B-2007-627-309), and each participant provided written informed consent.

### 2.2. Assessment of Dietary Intake

Dietary intake was assessed using 3-day food records. Energy and nutrient intake for each participant was calculated based on the Korean Food Composition Table, ninth revision, which was developed by the Korean National Rural Resources Development Institute [28]. The proportion of energy from carbohydrate, protein, and fat and the ratios of PUFA to SFA (PUFA/SFA), monounsaturated fatty acids (MUFA) to SFA (MUFA/SFA), iron to PUFA (iron/PUFA), iron to omega-6 PUFA (iron/omega-6 PUFA), and iron to omega-3 PUFA (iron/omega-3 PUFA) were also calculated.

### 2.3. Assessment of DPN

DPN was assessed using the Michigan Neuropathy Screening Instrument (MNSI), which includes two separate assessments: a 15-item, self-administered questionnaire (MNSI-Q) and a lower-extremity physical examination (MNSI-PE) [29]. The MNSI was validated in individuals with type 2 diabetes in Korea [30]. Trained healthcare providers performed all neuropathic examinations, and participants were diagnosed as having DPN when their MNSI-PE score was ≥2.5.

### 2.4. Anthropometric and Biochemical Measurements

Anthropometric indices were measured in barefoot participants wearing light clothing by a trained research nurse. Body mass index (BMI) was calculated as weight (kg) divided by the square of the height (m). Systolic and diastolic blood pressure (BP) were measured using an electronic BP measuring device after 10 min rest in a sitting position. We defined drinkers as those who drink any alcoholic beverage more than once a month. Smoking status was classified as never smoker (<100 cigarettes in lifetime and currently a nonsmoker), ex-smoker (≥100 cigarettes in lifetime and currently a nonsmoker), and current smoker (≥100 cigarettes in lifetime and currently a smoker). Blood samples were collected after an overnight fast. Plasma glucose levels were measured by the hexokinase method, and glycated hemoglobin (HbA_1c_) levels were measured by high-performance liquid chromatography (Bio-Rad, Hercules, CA, USA). Serum insulin levels were measured by an immunoradiometric assay (DIAsource ImmunoAssays, Nivelles, Belgium). Total cholesterol, triglycerides, high-density lipoprotein (HDL)–cholesterol and low-density lipoprotein (LDL)–cholesterol were measured by an enzymatic colorimetric assay. Liver functions tests, renal function tests, serum transferrin, ferritin, iron, total iron-binding capacity (TIBC), and neuron-specific enolase (NSE) were measured using established protocols by the Central Laboratory of SNUBH. Homeostatic model assessment for insulin resistance (HOMA-IR) and homeostatic model assessment for beta-cell function (HOMA-B) were calculated using the approximation equation of Matthews et al. [31]. Serum hepcidin and nitrotyrosine were measured using commercial enzyme-linked immunosorbent assay (ELISA) kits (Intrinsic Life Sciences, La Jolla, CA, USA; Hycult Biotech, Uden, The Netherlands).

### 2.5. Statistical Analysis

Data were expressed as mean ± standard deviation (SD) or number (%). Variables that were not normally distributed were natural log-transformed prior to analysis. Comparisons of continuous variables between individuals with DPN and those without DPN were performed using Student’s unpaired *t*-tests. Categorical variables were compared using chi-square tests. Nutrient intakes of individuals with and without DPN were compared by analysis of covariance with adjustment for total energy intake. After adjustment for total energy intake, Pearson’s correlation coefficient and partial correlation coefficient were used to evaluate the correlations between MNSI-PE scores and the mean daily intakes of energy and nutrients. The association of dietary iron intake, iron/PUFA ratio, iron/omega-6 PUFA ratio, and iron/omega-3 PUFA ratio with DPN was analyzed using logistic regression models. Multivariable logistic regression analyses were performed using significantly different variables between individuals with DPN and those without DPN and known risk factors for DPN. In all cases, *p* < 0.05 was considered statistically significant. Statistical analyses were performed using SPSS 25.0 (IBM, SPSS, Armonk, NY, USA).

## 3. Results

### 3.1. Characteristics of the Participants

The present study analysis included data from 147 of 200 individuals with type 2 diabetes, while the remaining 53 individuals did not complete their three-day food records. There were no significantly different demographic or biochemical data between individuals included and excluded in the analysis (Table S1). Among the participants, 46.3% were diagnosed as having DPN. There was no difference in sex or age between individuals with or without DPN. BMI and HOMA-IR were numerically higher in individuals with DPN than in those without DPN, but the difference was not significant. The eGFR was lower in individuals with DPN than in those without DPN. Total cholesterol and LDL–cholesterol levels were lower in individuals with DPN than in their counterparts without DPN. There was no difference in the frequency of usage of lipid-lowering drugs, but the percentage of moderate-intensity statin users was numerically higher in individuals with DPN than in those without DPN (Table 1, Table S2). The frequency of antidiabetic drugs’ use, smoking, and alcohol consumption was comparable between groups (Table 1, Table S3).

### 3.2. Iron Intake, Iron/PUFA Ratio, and DPN

Total energy intake and the percentage of energy from carbohydrates, protein, and fat were comparable between individuals with DPN and those without DPN. After adjustment for total energy intake, iron intake was significantly higher in individuals with DPN (10.9 ± 4.0 mg vs. 9.9 ± 3.6 mg, *p* = 0.041). Intakes of total PUFA, omega-6 PUFA, and omega-3 PUFA and the omega-6/omega-3 PUFA ratio were comparable between groups. Interestingly, the iron/PUFA ratio was significantly higher in individuals with DPN (1.4 ± 0.8 vs. 1.1 ± 0.4, *p* = 0.005). In addition, both iron/omega-6 PUFA ratio and iron/omega-3 PUFA ratio were significantly higher in individuals with DPN (Table 2).

MNSI-PE scores were positively correlated with iron intake after adjustment for total energy intake (*r* = 0.262, *p* = 0.001) and with the iron/PUFA ratio (*r* = 0.276, *p* = 0.001) (Table 3). In addition, MNSI-PE scores were also positively correlated with the iron/omega-6 PUFA ratio and the iron/omega-3 PUFA ratio. However, other nutrients did not display any significant correlation with MNSI-PE scores. After adjustment for total energy intake, sex, age, BMI, systolic BP, diabetes duration, eGFR, HbA_1c_, LDL–cholesterol, and smoking, logistic regression analyses showed that iron intake was associated with DPN (OR: 1.152; 95% CI: 1.012, 1.311). However, the level of significance was diminished after adjustment for HOMA-IR or HOMA-B. After full adjustment, the iron/PUFA ratio and the iron/omega-6 PUFA ratio were consistently associated with DPN, but the iron/omega-3 PUFA ratio was not significantly associated with DPN (Table 4).

### 3.3. Biochemical Markers of Iron Status and Oxidative Stress Markers of DPN

Biochemical markers of iron status such as serum transferrin, ferritin, iron, TIBC, and hepcidin were comparable between individuals with and those without DPN. The levels of oxidative stress markers of DPN, such as NSE and nitrotyrosine, were not different between individuals with and without DPN (Table 5).

## 4. Discussion

In this cross-sectional study, we observed that iron intake and iron intake relative to PUFA levels were higher in participants with DPN than in participants without DPN. Furthermore, the presence of DPN or the severity of DPN assessed by the MNSI-PE was positively associated with iron intake and the iron/PUFA ratio.

Prospective cohort studies of the Japanese and Chinese populations reported that iron intake was associated with an increased risk of diabetes [20,32]. In a case–control study of Europids with type 2 diabetes, a hemochromatosis-causing mutation C282Y was associated with a higher risk of diabetic retinopathy [33]. In a rat model, iron caused renal tubular injury due to the formation of free hydroxyl radicals [34]. A prospective intervention study revealed that a low-iron diet delayed the progression of diabetic nephropathy [35]. In regard to neuropathy, an in vitro study demonstrated that iron overload aggravated the oxidative stress injury of neurons in the presence of high glucose concentrations [36]. In our study, iron intake was associated with DPN, and this association was no longer significant after adjustment for HOMA-IR or HOMA-B. Therefore, insulin resistance and pancreatic beta cell dysfunction might be an important factor promoting the association between iron and DPN.

Oxidative stress causes pancreatic beta cell dysfunction, insulin resistance, and diabetic complications [37,38]. Meanwhile, iron can generate oxidative stress by the formation of hydroxyl radicals through the Fenton reaction [39]. Therefore, we need to measure reactive oxygen species (ROS) to understand the possible mechanism underlying the role of iron in DPN. However, the measurement of ROS is very tricky due to their reactivity and unstable properties [40]. Therefore, it might be more reasonable to measure oxidation target products of ROS. Previous cross-sectional studies reported that serum NSE and nitrotyrosine levels, which are oxidation target products of ROS, were closely associated with DPN [41,42]. In this background, we tested whether these indices were associated with DPN and useful for identifying DPN. However, we did not observe any differences in the levels of these biomarkers between individuals with DPN and those without DPN. This negative result might relate to the characteristics of the study subjects. We enrolled participants with relatively well-controlled type 2 diabetes, in contrast to the earlier study which enrolled subjects with both type 1 and type 2 diabetes [41], and with a more severe degree of hyperglycemia; the mean HbA_1c_ was up to 9.4% [42]. In fact, there are other oxidative markers of ROS-induced modifications of lipids, proteins, and DNA or RNA that we need to measure further [43]. For example, serum malondialdehyde and urinary 8-hydroxy-2′-deoxy-guanosine might be good candidates, because their levels were shown to be higher in individuals with diabetic nephropathy [44] and in individuals with diabetic microvascular complications [45]. However, we did not have available samples to test these molecules, which is one of the limitations of our study.

Hepcidin is an established master regulator of iron metabolism and an index of the iron pool in the body [46] that predicted the progression of diabetic nephropathy, one of the microvascular complications of type 2 diabetes [47]. However, in the present study, we did not observe differences in the levels of serum transferrin, ferritin, iron, and hepcidin linked to the presence of DPN. Therefore, we cautiously infer that a dietary pattern including high-iron-containing food might be a more important risk factor for DPN than the actual amount of iron. In addition, there are two types of dietary iron: heme and non-heme iron. Heme iron is present in red meat, poultry, and seafood, while non-heme iron is present in both plant and animal foods. Heme iron contributes 10–15% of total iron intake, but because of its higher absorption, it can contribute over 40% of the total absorbed iron [48]. As it is thought that the gut microbiota can influence the absorption capacity of iron [49], it might be necessary to consider both the amount and the quality of iron intake, as well as gut environmental factors, to best assess iron absorption.

Our results are different from results of rodent models of DPN which showed that iron deficiency rather than iron overload was associated with the risk of DPN [21,22,23]. It is hard to compare the results from human and rodent studies. In addition, cross-sectional studies and the intervention studies are different in terms of interpreting cause and effect. Even after consideration of the aforementioned points, we suggest that following might lead to different results in rodent models of DPN. First, in streptozotocin-diabetic rats, a single high dose of streptozotocin could induce nonspecific toxicity, which affects neurons directly [50]. In that circumstance, iron deficiency might cause impairment of iron-containing repair enzymes. Second, in *ob/ob* and *db/db* mice, iron deficiency can cause iron-deficiency anemia. The hemoglobin level in *db/db* mice on a high-iron diet was 19.4 g/dL, while it was 10.7 g/dL in *db/db* mice on a low-iron diet. This difference in hemoglobin might cause ischemia in the peripheral limbs. In contrast, considering that the individuals in our study did not have iron-deficiency anemia, iron deficiency might not have influenced our study results.

PUFA can be divided into two subclasses: omega-6 and omega-3. Omega-6 PUFA include linoleic acid and arachidonic acid [51]. Omega-3 PUFA include alpha-linolenic acid, eicosapentaenoic acid, and docosahexaenoic acid [52]. Omega-3 PUFA is an antioxidant able to produce a direct superoxide scavenging effect [24] and an indirect reactive oxygen species reduction effect via upregulation of antioxidant molecules [53]. Previous cross-sectional studies demonstrated that PUFA intake was associated with a lower odds ratio (OR) for the presence of diabetic retinopathy [54] and that linolenic acid intake was associated with lower odds of peripheral neuropathy [27]. A meta-analysis revealed that omega-3 fatty acid supplementation reduced the amount of proteinuria in individuals with type 2 diabetes [55]. For the cardiovascular risk, a few randomized controlled trials [56,57] and a meta-analysis [58] did not show a benefit of omega-3 PUFA, but the Japan EPA Lipid Intervention Study (JELIS) [59] and the Reduction of Cardiovascular Events with Icosapent Ethyl-Intervention Trial (REDUCE-IT) [60] have shown a benefit of omega-3 PUFA [60]. Until now, there is controversy about the role of omega-3 PUFA supplements for individuals with diabetes in the prevention of cardiovascular events [7]. To our best knowledge, there is a lack of studies investigating the role of omega-3 PUFA supplements for DPN. Considering the high prevalence of DPN and the limited treatment options for DPN, it is valuable to investigate the association between omega-3 PUFA and the risk of DPN. In this study, we observed a lower trend of PUFA and omega-3 PUFA intake in individuals with DPN compared to those without DPN. Meanwhile, omega-6 PUFA is considered pro-inflammatory by some researchers because linoleic acid, the representative of omega-6 PUFA, is converted into arachidonic acid. In addition, a few studies suggested that omega-6 PUFA is related to chronic inflammatory diseases such as obesity, nonalcoholic fatty liver disease, cardiovascular disease [61,62]. However, other studies showed that high consumption of omega-6 PUFA did not increase cardiovascular events [63,64]. In this study, we observed a lower, but not significant, omega-6 PUFA intake in individuals with DPN. Considering the significant correlation between omega-6 PUFA intake and omega-3 PUFA intake in this study (*r* = 0.713, *p* < 0.001), it is not possible to interpret the results of omega-6 PUFA and omega-3 PUFA separately.

**Figure 1:**
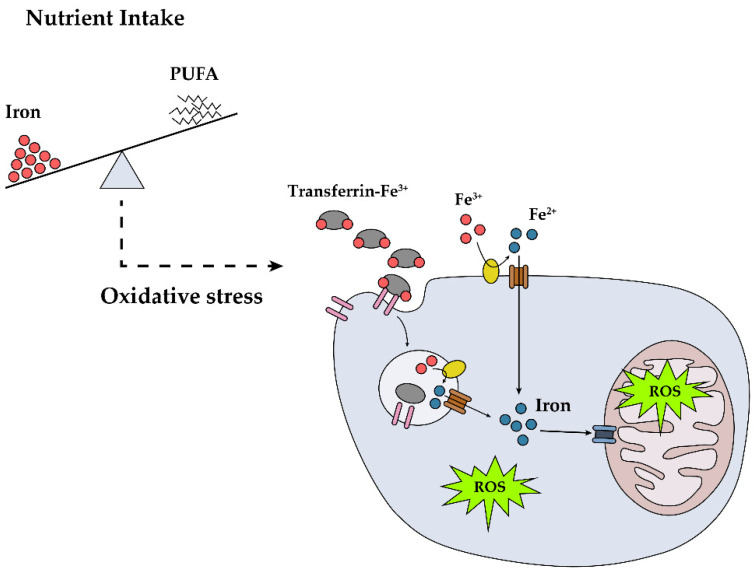
Potential role of dietary iron in the development of diabetic peripheral neuropathy (DPN). High dietary iron and iron intake relative to polyunsaturated fatty acids (PUFA) lead to DPN through reactive oxygen species (ROS) formation.

In light of the in vitro [36] and animal model [65] data, we postulated that increased body iron can damage neurons or Schwann cells via direct or indirect pathways. Considering the PUFA-related antioxidant effect observed in an iron-related, pro-oxidant environment, we calculated the iron/PUFA ratio and found that a higher iron/PUFA ratio was associated with a higher OR of DPN. This finding suggests that the ratio of iron to PUFA might be an important marker of DPN (Figure 1) and can be used as an indicator to screen for or prevent DPN in individuals with type 2 diabetes. In addition, even though the ratio iron/omega-6 PUFA, rather than the ratio iron/omega-3 PUFA, showed a statistically significant association with DPN after adjusting for confounders, we need to be cautious in interpreting these data. A relatively small amount of omega-3 PUFA compared with omega-6 PUFA might bring about these non-significant results.

Our study has several limitations. First, because of its cross-sectional nature, we could not establish a causal relationship. Second, our study was based on a relatively small sample size, which may have affected the assessment of significant differences in known risk factors for DPN, such as age, BMI, and diabetes duration. Studies with a larger sample size or a prospective or intervention study would be of interest to confirm or reassess these findings. Third, neurophysiologic studies were not used to confirm the DPN diagnosis. Fourth, the Korean Food Composition Table does not contain data regarding haem iron content, so we could not analyze the intake of heme iron and non-heme iron separately. Lastly, among various oxidative stress markers, we measured only NSE and nitrotyrosine levels, which were comparable between groups.

Despite these limitations, this study has several strengths. First, we obtained dietary nutrient intake estimates with the use of three-day food records. In addition, we controlled for a number of dietary and nondietary covariates to reduce possible confounding effects. Above all, this is the first study to examine the association between dietary iron intake and DPN. In addition, we suggest the iron/PUFA ratio as a new index associated with DPN.

## 5. Conclusions

Dietary iron intake and the iron/PUFA ratio were associated with DPN. The present study suggests the importance of the dietary pattern of iron and PUFA intake in individuals with type 2 diabetes, which might be an intervention target for preventing or treating DPN.

## Figures and Tables

**Table 1:** Clinical and biochemical data of individuals according to the presence of diabetic peripheral neuropathy (DPN).

Characteristic	DPN (–) (*n* = 79)			DPN (+) (*n* = 68)			*p* Value
Male, *n* (%)	45 (56.9)			41 (60.3)			0.683
Age (years)	57.5	±	9.0	60.1	±	9.8	0.090
Height (cm)	163.4	±	8.1	163.3	±	9.2	0.938
Body weight (kg)	66.1	±	11.0	68.7	±	11.1	0.158
BMI (kg/m^2^)	24.7	±	3.2	25.7	±	2.9	0.053
Systolic BP (mmHg)	128	±	13	132	±	15	0.155
Diastolic BP (mmHg)	75	±	9	75	±	9	0.971
Diabetes duration (years)	9.4	±	7.2	10.4	±	7.0	0.377
FPG (mmol/L)	7.5	±	1.6	7.9	±	2.3	0.222
HbA_1c_ (mmol/mol)	54.6	±	13.1	57.9	±	15.3	0.154
HbA_1c_ (%)	7.1	±	1.2	7.4	±	1.4	0.153
Total cholesterol (mmol/L)	4.3	±	1.0	3.9	±	0.8	0.002
Triglyceride (mmol/L) ^a^	1.3	±	0.0	1.4	±	0.0	0.283
HDL–cholesterol (mmol/L) ^a^	1.2	±	0.0	1.2	±	0.0	0.070
LDL–cholesterol (mmol/L)	2.5	±	0.7	2.2	±	0.6	0.006
Urea nitrogen (mmol/L)	5.6	±	1.5	5.9	±	2.3	0.378
Creatinine (μmol/L)	68.1	±	18.6	74.3	±	23.0	0.094
eGFR (mL min^−1^ (1.73 m)^−2^)	97.4	±	21.1	90.1	±	22.2	0.042
AST (U/L)^a^	26.0	±	1.4	27.2	±	1.4	0.437
ALT (U/L)	28.9	±	17.1	27.1	±	12.3	0.469
Insulin (pmol/L)	58.1	±	32.2	63.9	±	33.0	0.293
HOMA-IR	2.6	±	1.6	3.2	±	1.9	0.065
HOMA-B ^a^	38.8	±	2.3	40.9	±	2.0	0.670
MNSI-Q (score)	1.7	±	2.0	2.9	±	2.1	<0.001
MNSI-PE (score)	1.3	±	0.6	3.5	±	0.7	<0.001
Smoking status, *n* (%)							0.350
Never smoker	33 (41.8)			36 (52.9)			
Ex-smoker	30 (38.0)			19 (27.9)			
Current smoker	16 (20.3)			13 (19.1)			
Alcohol, *n* (%)	43 (54.4)			34 (50.0)			0.592

Data are expressed as mean ± SD or geometric mean ± geometric SD or number (%). ^a^ Variable was natural log-transformed before statistical analysis and expressed as geometric mean ± geometric SD. DPN, diabetic peripheral neuropathy; BMI, body mass index; BP, blood pressure; HbA_1_, glycated hemoglobin; HDL, high-density lipoprotein; LDL, low-density lipoprotein; eGFR, estimated glomerular filtration rate; AST, aspartate aminotransferase; ALT, alanine aminotransferase; HOMA-IR, homeostatic model assessment for insulin resistance; HOMA-B, homeostatic model assessment for beta cell function; FPG, fasting plasma glucose; MNSI-Q, Michigan Neuropathy Screening Instrument-questionnaire; MNSI-PE, Michigan Neuropathy Screening Instrument-physical examination.

**Table 2:** Mean daily intake of energy and nutrients estimated from three-day food records according to the presence of DPN.

Variable	DPN (–) (*n* = 79)			DPN (+) (*n* = 68)			*p* Value	*p* Value ^a^
TE intake (kJ)	6255.9	±	1812.5	6218.3	±	1412.6	0.889	NA
Carbohydrate (% TE)	61.5	±	12.2	63.4	±	10.7	0.327	NA
Protein (% TE)	16.5	±	3.5	16.5	±	3.2	0.934	NA
Fat (% TE)	23.7	±	9.2	22.9	±	9.3	0.612	NA
SFA (g)	13.4	±	8.1	12.6	±	7.1	0.500	0.434
MUFA (g)	13.0	±	7.9	13.0	±	7.9	0.987	0.887
PUFA (g)	10.2	±	5.2	9.2	±	4.7	0.211	0.155
Omega-6 PUFA (g)	8.6	±	4.5	7.6	±	3.9	0.144	0.092
Omega-3 PUFA (g)	1.4	±	0.8	1.3	±	1.0	0.637	0.653
Omega-6/Omega-3 PUFA ratio	7.2	±	2.7	7.3	±	3.1	0.811	NA
PUFA/SFA ratio	0.9	±	0.6	0.9	±	0.5	0.698	NA
MUFA/SFA ratio	1.0	±	0.2	1.0	±	0.2	0.253	NA
Iron (mg)	9.9	±	3.6	10.9	±	4.0	0.130	0.041
Iron/PUFA ratio (mg/g)	1.1	±	0.4	1.4	±	0.8	0.005	NA
Iron/omega-6 PUFA ratio (mg/g)	1.3	±	0.5	1.7	±	1.1	0.006	NA
Iron/omega-3 PUFA ratio (mg/g)	9.3	±	5.5	12.1	±	7.8	0.016	NA
Vitamin B1 (mg)	0.9	±	0.4	0.8	±	0.3	0.635	0.625
Vitamin B6 (mg)	0.4	±	0.2	0.4	±	0.2	0.922	0.871
Vitamin B12 (µg)	3.7	±	3.1	3.7	±	3.5	0.988	0.967
Vitamin D (µg)	4.2	±	4.2	3.1	±	4.0	0.116	0.118
Dietary fiber (g)	21.7	±	8.2	23.3	±	8.1	0.240	0.156

Data are expressed as mean ± SD. ^a^ *p* value for ANCOVA adjusted for total energy intake. DPN, diabetic peripheral neuropathy; TE, total energy; NA, not applicable; SFA, saturated fatty acids; MUFA, monounsaturated fatty acids; PUFA, polyunsaturated fatty acids; PUFA/SFA, PUFA intake to SFA intake; MUFA/SFA, MUFA intake to SFA intake.

**Table 3:** Correlation analysis between MNSI-PE scores and mean daily intake of energy and nutrients.

Variable	Coefficient	*p* Value
Carbohydrate (% TE)	0.095	0.250
Protein (% TE)	0.014	0.870
Fat (% TE)	−0.059	0.480
SFA (g) ^a^	−0.078	0.347
MUFA (g) ^a^	−0.037	0.661
PUFA (g) ^a^	−0.118	0.155
Omega-6 PUFA ^a^	−0.146	0.079
Omega-3 PUFA ^a^	0.003	0.976
Omega-6/Omega-3 PUFA ratio	−0.023	0.780
PUFA/SFA ratio	0.003	0.969
MUFA/SFA ratio	0.029	0.731
Iron (mg) ^a^	0.262	0.001
Iron/PUFA ratio (mg/g)	0.276	0.001
Iron/omega-6 PUFA ratio (mg/g)	0.271	0.001
Iron/omega-3 PUFA ratio (mg/g)	0.204	0.013
Vitamin B1 (mg) ^a^	−0.075	0.368
Vitamin B6 (mg) ^a^	0.021	0.806
Vitamin B12 (µg) ^a^	0.056	0.501
Vitamin D (µg) ^a^	−0.089	0.285
Dietary fiber (g) ^a^	0.151	0.068

Pearson’s correlation analysis was conducted. ^a^ Partial correlation analysis was conducted after adjusting for total energy intake. MNSI-PE, Michigan Neuropathy Screening Instrument-physical examination; TE, total energy; SFA, saturated fatty acids; MUFA, monounsaturated fatty acids; PUFA, polyunsaturated fatty acids; PUFA/SFA, PUFA intake to SFA intake; MUFA/SFA, MUFA intake to SFA intake.

**Table 4:** Odds ratios (ORs, 95% CI) between iron intake, iron/PUFA, and DPN.

Variable	OR	95% CI	*p* Value
Iron intake			
Model 1	1.126	1.003, 1.264	0.044
Model 2	1.147	1.014, 1.298	0.029
Model 3	1.152	1.012, 1.311	0.032
Model 4	1.136	0.995, 1.297	0.059
Model 5	1.139	0.998, 1.301	0.053
Iron/PUFA ratio			
Model 1	2.628	1.324, 5.216	0.006
Model 2	2.375	1.168, 4.830	0.017
Model 3	2.283	1.066, 4.887	0.034
Model 4	2.215	1.032, 4.757	0.041
Model 5	2.214	1.034, 4.742	0.041
Iron/omega-6 PUFA ratio			
Model 1	2.321	1.287, 4.186	0.005
Model 2	2.136	1.160, 3.934	0.015
Model 3	2.096	1.089, 4.032	0.027
Model 4	2.037	1.058, 3.922	0.033
Model 5	2.046	1.063, 3.935	0.032
Iron/omega-3 PUFA ratio			
Model 1	1.069	1.012, 1.130	0.018
Model 2	1.051	0.992, 1.112	0.090
Model 3	1.054	0.995, 1.117	0.073
Model 4	1.055	0.995, 1.118	0.074
Model 5	1.052	0.993, 1.115	0.082

Model 1 is adjusted for total energy intake; Model 2 is additionally adjusted for sex, age, BMI, systolic BP, diabetes duration, and eGFR; Model 3 is additionally adjusted for HbA_1c_, LDL–cholesterol, and smoking; Model 4 is additionally adjusted for HOMA-IR from Model 3; Model 5 is additionally adjusted for HOMA-B from Model 3. PUFA, polyunsaturated fatty acids; DPN, diabetic peripheral neuropathy; BMI, body mass index; BP, blood pressure; eGFR, estimated glomerular filtration rate; HbA_1_, glycated hemoglobin; LDL, low-density lipoprotein; HOMA-IR, homeostatic model assessment for insulin resistance; HOMA-B, homeostatic model assessment for beta cell function.

**Table 5:** Laboratory biochemical data of individuals according to the presence of DPN.

Variable	DPN (–) (*n* = 79)			DPN (+) (*n* = 68)			*p* Value
Transferrin (μmol/L)	31.6	±	4.5	32.1	±	4.3	0.494
Ferritin (μg/L)	150.2	±	120.4	140.8	±	109.5	0.627
Iron (μmol/L)	19.2	±	7.0	19.2	±	6.8	0.975
TIBC (μmol/L)	60.6	±	8.2	61.9	±	7.9	0.325
TSAT (%)	31.9	±	10.5	31.7	±	11.9	0.897
Hepcidin (ng/mL)	3.2	±	1.7	3.0	±	1.7	0.525
NSE (ng/mL)	12.6	±	3.6	12.3	±	2.6	0.573
Nitrotyrosine (nmol/L) ^a^	10.0	±	2.2	9.0	±	2.0	0.276

Data are expressed as mean ± SD or geometric mean ± geometric SD. ^a^ Variable was natural log-transformed before statistical analysis and expressed as geometric mean ± geometric SD. DPN, diabetic peripheral neuropathy; TIBC, total iron-binding capacity; TSAT, transferrin saturation NSE, neuron-specific enolase.

## Supplementary Materials

The following are available online at https://www.mdpi.com/2072-6643/12/11/3365/s1, Table S1: Clinical and biochemical data of individuals according to the presence of DPN. Table S2: Use of lipid-lowering drugs in individuals according to the presence of DPN. Table S3: Use of antidiabetic drugs in individuals according the the presence of DPN.
