# Supplementary material for: Association between Iron Intake and Diabetic Peripheral Neuropathy in Type 2 Diabetes: Significance of Iron Intake and the Ratio between Iron Intake and Polyunsaturated Fatty Acids Intake
Source: Nutrients. 2020 Nov 1;12(11):3365. doi: 10.3390/nu12113365 (PMC7693683; doi:10.3390/nu12113365)
Supplement: Supplementary file 1 [file nutrients-12-03365-s001.pdf]

Table S1. Clinical and biochemical data of individuals according to the presence of DPN.

Characteristic	Study not enroll (n = 53)			Study enroll (n = 147)			p value
Male, n (%)	36 (67.9)			86 (58.5)			0.228
Age (years)	55.7	±	10.0	58.7	±	9.5	0.052
Height (cm)	165.1	±	9.8	163.4	±	8.6	0.227
Body weight (kg)	69.9	±	14.9	67.3	±	11.0	0.188
BMI (kg/m^2)	25.5	±	4.2	25.2	±	3.1	0.596
Systolic BP (mmHg)	128	±	19	130	±	14	0.368
Diastolic BP (mmHg)	76	±	10	75	±	9	0.635
Diabetes duration (years)	10.4	±	8.7	9.8	±	7.1	0.676
FPG (mmol/L)	8.1	±	2.5	7.7	±	2.0	0.154
HbA1c (mmol/mol)	59.7	±	17.5	56.1	±	14.0	0.184
HbA1c (%)	7.6	±	1.6	7.3	±	1.3	0.190
Total cholesterol (mmol/L)	4.2	±	1.2	4.1	±	0.9	0.571
Triglyceride (mmol/L) [a]	1.5	±	0.0	1.4	±	1.0	0.237
HDL cholesterol (mmol/L)	1.2	±	0.3	1.2	±	0.3	0.476
LDL cholesterol (mmol/L)	2.4	±	0.8	2.4	±	0.7	0.902
Urea nitrogen (mmol/L)	5.9	±	1.2	5.7	±	1.9	0.428
Creatinine (μmol/L)	70.7	±	17.7	70.7	±	17.7	0.282
eGFR (mL min^{-1} (1.73 m)$^{-2}$)	100.0	±	22.9	94.1	±	21.9	0.095
AST (U/L)	31.0	±	16.0	28.3	±	12.3	0.220
ALT (U/L)	30.8	±	20.8	28.0	±	15.1	0.372
Insulin (pmol/L)	57.6	±	24.3	59.0	±	31.3	0.793
HOMA-IR	3.0	±	1.8	2.9	±	1.6	0.576
HOMA-B [a]	37.6	±	1.8	39.2	±	2.1	0.737
MNSI-Q (score)	2.2	±	1.8	2.3	±	2.1	0.918
MNSI-PE (score)	2.6	±	1.3	2.3	±	1.3	0.099
Smoking status, n (%)							0.759
Never smoker	28 (52.8)			69 (46.9)			
Ex-smoker	16 (30.2)			49 (33.3)			
Current smoker	9 (17.0)			29 (19.7)			
Alcohol, n (%)	27 (50.9)			77 (52.4)			0.223

Data are expressed as mean ± SD or geometric mean ± geometric SD or number (%). [a] Variable was natural log-transformed before statistical analysis and expressed as geometric mean ± geometric SD. DPN, diabetic peripheral neuropathy; BMI, body mass index; BP, blood pressure; HbA_1, glycated hemoglobin; HDL, high-density lipoprotein; LDL, low-density lipoprotein; eGFR, estimated glomerular filtration rate; AST, aspartate aminotransferase; ALT, alanine aminotransferase; HOMA-IR, homeostatic model assessment for insulin resistance; HOMA-B, homeostatic model assessment for

beta cell function; FPG, fasting plasma glucose; MNSI-Q, Michigan Neuropathy Screening Instrument-questionnaire; MNSI-PE, Michigan Neuropathy Screening Instrument-physical examination.

Table S2. Use of lipid-lowering drugs in individuals according to the presence of DPN.

Variable	DPN (–) (*n* = 79)	DPN (+) (*n* = 68)	*p* value
Statin, *n* (%)			0.526
No statin	27 (34.2)	16 (23.5)	
Low-intensity statin	5 (6.3)	4 (5.9)	
Moderate-intensity statin	39 (49.4)	41 (60.3)	
High-intensity statin	8 (10.1)	7 (10.3)	
Ezetimibe, *n* (%)	4 (5.1)	8 (11.8)	0.226
Fenofibrate, *n* (%)	1 (1.3)	3 (4.4)	0.336

Data are expressed as number (%). DPN, diabetic peripheral neuropathy.

Table S3. Use of antidiabetic drugs in individuals according to the presence of DPN.

Variable	DPN (–) (*n* = 79)	DPN (+) (*n* = 68)	*p* value
Metformin, *n* (%)	71 (89.9)	59 (86.8)	0.557
Sulfonylurea, *n* (%)	19 (24.1)	21 (30.9)	0.353
DPP-4 inhibitor, *n* (%)	44 (55.7)	35 (51.5)	0.608
SGLT-2 inhibitor, *n* (%)	10 (12.7)	9 (13.2)	0.917
Thiazolidinedione, *n* (%)	6 (7.6)	5 (7.4)	0.956
α-glucosidase inhibitor, *n* (%)	1 (1.4)	1 (2.0)	0.797
GLP-1 agonist, *n* (%)	2 (2.5)	1 (1.5)	0.650
Insulin, *n* (%)	11 (13.9)	14 (20.6)	0.284

Data are expressed as number (%). DPN, diabetic peripheral neuropathy; DPP, dipeptidyl peptidase; SGLT, sodium-glucose cotransporter; GLP, glucagon-like peptide.
